# Age-specific uptake of non-invasive prenatal tests (NIPT) in Germany: a decision theory-based analysis

**DOI:** 10.1007/s12687-025-00822-2

**Published:** 2025-07-23

**Authors:** Michael Krawczak, Bernd Eiben, Sebastian Sendel, Amke Caliebe, Lidewij Henneman, Ralf Glaubitz, Heike Borth, Jörg Schmidtke

**Affiliations:** 1 Institut für Medizinische Informatik und Statistik, Christian-Albrechts- Universität zu Kiel, Universitätsklinikum Schleswig-Holstein, Brunswiker Straße 10, Kiel, 24105 Germany; 2 Institut für Klinische Genetik und Labormedizin Rhein/Ruhr GmbH MVZ, Essen, Germany; 3 Department of Human Genetics, Amsterdam Reproduction and Development Research Institute, Amsterdam UMC, location Vrije Universiteit, Amsterdam, The Netherlands; 4 MVZ amedes genetics für interdisziplinäre Labordiagnostik GmbH, Hannover, Germany; 5 amedes MVZ wagnerstibbe, Hannover, Germany; 6 Institut für Humangenetik, Medizinische Hochschule Hannover, Hannover, Germany

**Keywords:** Chromosomal aberration, Non-invasive prenatal testing, Maternal age, Counseling, Decision theory, Test costs

## Abstract

Non-invasive prenatal testing (NIPT) for fetal chromosomal aberrations is an important component of healthcare systems worldwide, albeit with varying diagnostic coverage and conditions of use. In Germany, NIPT primarily focuses on trisomies 21, 18 and 13, for which the test costs are reimbursed by the statutory health insurance after thorough prior counseling. Despite this rather restrictive approach compared to other countries, concerns continue to be raised in Germany that young pregnant women, in particular, who are at a low risk of fetal aneuploidy, may have been overly encouraged to undergo NIPT. However, a decision theory-based analysis of the NIPT uptake figures in Germany suggests that there is currently no evidence that avoiding the birth of a trisomic child is a strong motivation particularly of younger women to take the test. Instead, the nation-wide NIPT uptake figures are exceptionally well in line with the corresponding age-specific prior risks. Notably, no such agreement was found when we considered the Netherlands as an example of a healthcare system where NIPT covers additional chromosomal aberrations without age-dependent risk. Replication of our analysis in other countries will reveal whether a strong consistency between age-specific prior risk and NIPT uptake is unique to Germany, or not.

## Introduction

The 1997 discovery of cell-free fetal (placental) DNA in maternal plasma during pregnancy (Lo et al. 1997) paved the way for the development of novel means of prenatal testing for fetal aneuploidies, particularly trisomies 21, 18 and 13 (Lo et al. 2007). In 2011, the first commercial versions of this so-called ‘non-invasive prenatal test’ (NIPT) became available, soon to be followed by similar products also covering sex chromosomal aneuploidies (e.g., Gil et al. 2017), microdeletions (e.g., Bevilacqua et al. 2021), and other rare numerical and structural aberrations (e.g., van der Meij et al. 2019). The NIPT then rapidly spread around the world and, by 2021, was available in more than 60 countries, reaching a global annual market value of approximately 3 billion US Dollars (Ravitsky et al. 2021).

In Germany, first-trimester screening by nuchal translucency measurement and biochemical analyses became available in the early 2000s, but had to be paid for by the pregnant woman out-of-pocket. From 2012 onwards, these early tests were increasingly supplemented by NIPT and, in 2019, the Gemeinsamer Bundesausschuss as the responsible national authority decided that NIPT may be reimbursed by the statutory health insurance if the test was targeted exclusively at the three above-mentioned trisomies (Gemeinsamer Bundesausschuss 2021). This restrictive approach differs significantly from that taken in other countries where comprehensive testing of a wide range of chromosomal aberrations constitutes the test portfolio. Moreover, NIPT may only be reimbursed in Germany if offered on a case-by-case basis following individual counselling, and not as a way of second-tier screening of high-risk pregnancies or first-tier screening of all pregnancies (Gemeinsamer Bundesausschuss 2021). This restriction was probably due to concerns that, if NIPT was offered routinely as an unconditionally publicly funded service, it might be seen as officially recommended and would therefore place undue pressure on pregnant women to take the test (Bowman-Smart et al. 2023).

The above considerations are also reflected in the current political debate on NIPT in Germany. In a 2024 motion to the Deutscher Bundestag, the German federal parliament, that called for monitoring of the consequences of statutory NIPT reimbursement (Deutscher Bundestag 2024), 120 parliament members expressed concerns that the test is recommended to pregnant women without a so-called ‘medical indication’ which, in Germany, almost exclusively covers women of advanced age. The authors of the motion even suggested that this practice reflects the desire of physicians to protect themselves from liability for medical malpractice. Regardless of the validity of this allegation, intensive counselling before NIPT clearly places strong emphasis upon the consequences of taking or not taking the test. Such a strong focus on the issue may indeed have led to women, especially younger women, being tested more frequently than was reasonable given their age-specific prior risk. These worries could have been exacerbated further by the fact that the NIPT uptake among young pregnant women has indeed increased worldwide (Sebire et al. 2024).

If the above concerns were justified, and if maternal age played a minor role in counselling and subsequent decision-making about NIPT in Germany, then the national uptake figures of the test should have somehow levelled out across age groups. Recently, the first comprehensive data on the uptake of NIPT in Germany after its recognition as a reimbursable service were published by Barmer Institut für Gesundheitssystemforschung (bifg), an independent research arm of Barmer which is one of the largest statutory health insurance funds in the country (Barmer Institut für Gesundheitsforschung 2024). We subjected these data to a decision theory-based analysis to determine whether or not the relationship between NIPT uptake and age-dependent aneuploidy risk is consistent across age groups in Germany.

## Methods

According to classical decision theory (Melsa and Cohn 1978), a rational decision to undergo NIPT would be based upon (i) the material and immaterial costs that a pregnant woman and their family assign to having a trisomic child (C_T_) and to taking the test (C_N_), and (ii) the individual trisomy risk r. Worthy of note, C_N_ not only includes the monetary costs and socio-ethical demands of the test itself, but also accounts for the possibly invasive follow-up of a positive test result. Furthermore, taking the individual trisomy risks of pregnant women into account in our considerations only makes sense if these risks actually play an important role in their decision-making. While this can be assumed to hold true in Germany, it may not be the case in countries where NIPT is implemented, for example, as a first-stage screening test of all pregnancies, regardless of maternal age.

Given the above, so-called ‘Bayes decision-making’ (Ma 2019) would imply that a decision to take or not take the test is made in a way that minimizes the expected costs associated with a wrong decision, i.e. of (i) not taking the test in an affected pregnancy, or of (ii) taking the test in a non-affected pregnancy. For NIPT, this means that the test is taken if, and only if,

1 $$ \text{r} \cdot \text{C}_{\text{T}} > \text{C}_{\text{N}}, \text{which is equivalent to}\, \text{C}_{\text{T}}/\text{C}_{\text{N}} = \text{C} > \text{r}^{-1}, $$

i.e. if the pregnant woman and her family perceive the birth of a trisomic child r^− 1^ times more burdensome than the test itself.

Since the relative costs C assigned to having a trisomic child rather than undergoing NIPT certainly vary between cases, it is reasonable to assume that C is a random variable following an N(µ,σ) normal distribution, where µ and σ reflect the personal context of the decision-makers (e.g., the age of the pregnant woman). Let c_α_ denote the α-quantile of a N(µ,σ) distribution, i.e. P(C ≤ c_α_)=α for 0 < α < 1. Since random variable (C-µ)/σ has an N(0,1) distribution, it follows that

2 $$ (\text{c}_{\alpha} - \mu)/\sigma = \Phi^{-1}(\alpha), $$

where Φ is the distribution function of the N(0,1) distribution. This implies that, given a specific value of α, linear relationship

3 $$ \mu = \text{c}_{\alpha} - \Phi^{-1}(\alpha)\cdot{\sigma} $$

holds for all combinations of µ and σ for which the α-quantile of an N(µ,σ) distribution equals c_α_. Now, if α is set equal to 1-q, where q denotes the probability of undergoing NIPT, formula (3) implies that c_α_ can be replaced by r^− 1^ in formula (3), which leads to

4 $$ \mu = \text{r}^{-1} - \Phi^{-1}(1 - \text{q})\cdot{\sigma}. $$

In other words, if decision-making about NIPT follows the logic epitomized by formula (1), the uptake of the test in a particular age group allows some conclusions to be drawn about the perception in that group of the burden of a trisomic child. Although the mean and variance of C themselves cannot be determined, their (linear) relationship is unambiguous and thus characterizes the respective attitude of the group.

## Results

According to bifg (see Introduction), the 2019–2023 uptake of NIPT among pregnant women in Germany was 25% up to the age of 25 years (group 1, q_1_ = 0.25), 50% between 26 and 35 years of age (group 2, q_2_ = 0.50), and 70% among woman aged 36 years or older (group 3, q_3_ = 0.70) (Barmer Institut für Gesundheitsforschung 2024). In 2023, these age groups accounted for 12.6%, 63.5% and 23.9% of all pregnancies in Germany, respectively (Statista 2025). The corresponding aneuploidy risks roughly equal r_1_ = 10^− 3^, r_2_ = 2 × 10^− 3^, and r_3_ = 10^− 2^ (Institut für Qualität und Wirtschaftlichkeit im Gesundheitswesen 2021).

Unfortunately, bifg published their uptake figures only in this relatively rough breakdown so that a more precise analysis of the age relationship is not possible at this stage. Anyhow, since

5 $$ \begin{aligned} \Phi^{-1}(1 - \text{q}_{1}) & = \Phi^{-1}(0.75) = 0.67, \text{r}_{1}^{-1} = 1000 \\ \Phi^{-1}(1 - \text{q}_{2}) & = \Phi^{-1}(0.50) = 0.00, \text{r}_{2}^{-1} = 500 \\ \Phi^{-1}(1 - \text{q}_{3}) & = \Phi^{-1}(0.30) = -0.53, \text{r}_{3}^{-1} = 100 \end{aligned} $$

as derived from the tabulated quantiles of the N(0,1) distribution or by inversion of the aneuploidy risks, respectively, the following linear relationships hold between the respective values of µ_i_ and σ_i_:

6 $$ \begin{aligned} \mu_{1} &= 1000 - 0.67 \cdot \sigma_1 \\ \mu_{2} &= 500 \\ \mu_{3} &= 100 + 0.53 \cdot \sigma_3 \end{aligned} $$

**Fig. 1:**
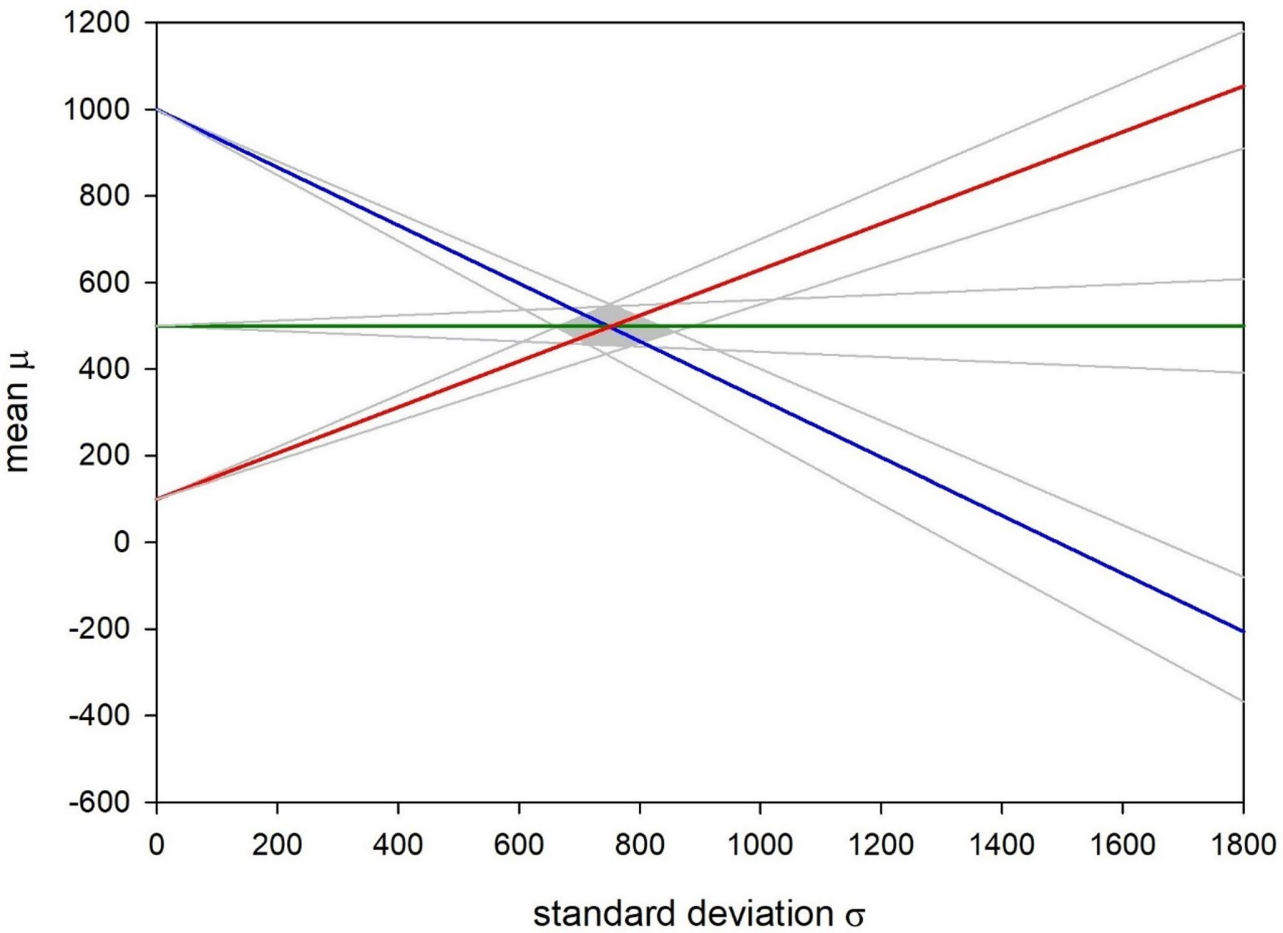
Inferred age-specific relationship between the mean (µ) and standard deviation (σ) of the relative trisomy costs (C) assigned by German women. Blue: age group 1; green: age group 2, red: age group 3, grey lines: relationships inferred for modified (± 2.5%) age-specific NIPT uptake figures, grey area: location of pair-wise intersection points if one or more age-specific uptake figure varied by up to 2.5%

This means that, in age group 2, the average relative costs μ assigned to the birth of a trisomic child equal 500 (i.e., this event is considered 500 times more severe than the test itself), regardless of the actual variance σ of the costs. In the other two age groups, by contrast, µ changes with σ. Notably, the three lines defined by formulas (6) intersect almost perfectly at a single point (Fig. 1), which is as expected if the relative cost assignments were the same in the three age groups (i.e., if µ_1_=µ_2_=µ_3_ and σ_1_ = σ_2_ = σ_3_). If this hypothesis were wrong, intersection at a single point by chance alone would be very unlikely.

Strictly speaking, it cannot be excluded that the uptake figures reported by bifg (Barmer Institut für Gesundheitsforschung 2024) were rounded to the nearest 5% (i.e., modified by up to ± 2.5%), before publication. However, even when such rounding error is accounted for, the resulting intersection points are still very close (grey area in Fig. 1). Following classical Bayesian logic, the posterior odds for an (almost) identical relative cost assignment in the three age groups are thus extremely high, provided that the prior odds are not extremely low. Thus, even if prior counseling on NIPT led to an increase of C among German women, this change must have been the same across all age groups.

## Discussion

After initial misunderstandings regarding the performance of the NIPT could be cleared up (Eiben et al. 2024), the main criticism of the test in Germany is now that it is recommended to pregnant women regardless of medical relevance. These concerns relate primarily to younger women, who are allegedly prescribed NIPT despite a low prior risk of the three aneuploidies eligible for statutory reimbursement. However, our results suggest that there is currently no evidence of a specifically (and unjustly) increased tendency of younger pregnant woman in Germany to undergo NIPT for trisomies 21, 18 and 13. Therefore, any allegations that women are pressured by their doctors to do so are most likely unjustified. The latter conclusion is further supported by a recent survey in Germany (von Ostrowsky et al. 2025) in which only 6% of respondents reported that they felt influenced by their doctor in their decision-making for or against NIPT.

A recent study confirmed that, while NIPT has gained global acceptance, its national uptake figures vary widely depending upon previous and current screening practices, the actual design of the test (e.g., first-tier or second-tier), its out-of-pocket costs, and its cultural and health policy framework. Consequently, the NIPT uptake may be as low as 1.5% in Ontario, Canada (first-tier, women ≥ 40 years), and as high as 93.2% in Andalusia, Spain (second-tier, high-risk pregnancies only) (Sebire et al. 2024). To validate the decision theory-based approach of our work, it would thus be helpful to compare NIPT uptake in Germany with that in countries where comprehensive NIPT is offered as a nationwide screening not limited to aberrations with age-dependent risk.

**Fig. 2:**
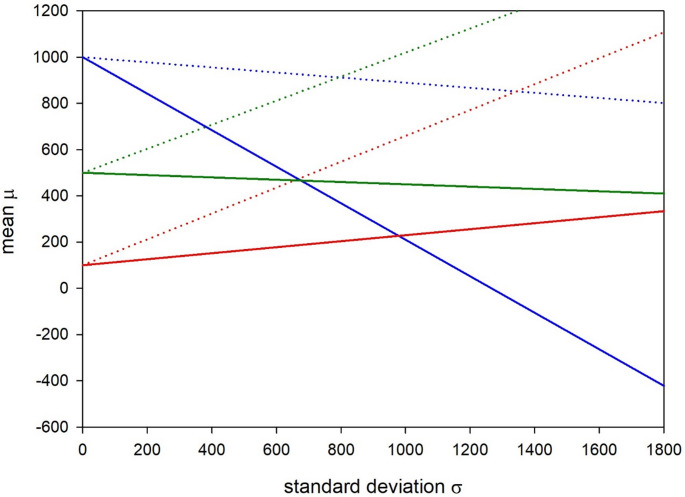
Inferred age-specific relationship between the mean (µ) and standard deviation (σ) of the relative trisomy costs (C) assigned by Dutch women. Solid lines: 2019 data, dotted lines: 2023 data. For details, see legend to Fig. 1

In the Netherlands, NIPT was implemented from the outset as a national screening program that covers trisomies 21, 18 and 13, but that also targets other numerical and structural chromosomal aberrations, depending upon the women’s wishes (van der Meij 2019). The first age-specific NIPT uptake figures were reported for the Netherlands, in 2019, to equal q_1_ = 0.21, q_2_ = 0.48, and q_3_ = 0.55 in neighborhoods that were not specifically disadvantaged (van der Meij et al. 2021). With these numbers, the theoretically inferred cost assignments would closely resemble those of German women in age groups 1 and 2 (Fig. 2). Above the age of 35 years, however, the slope of the corresponding (red) line was substantially smaller for Dutch women than for German women of today, apparently indicating lower relative costs C assigned to delivering and raising a trisomic child. Worthy of note, monetary test costs in the Netherlands had to be partly covered out-of-pocket in 2019 (at 175 Euros), which could have increased C_N_ and decreased C in age group 3 as well.

In any case, by 2023, the NIPT uptake figures in the Netherlands had increased to q’_1_=0.46, q’_2_=0.70, and q’_3_=0.71 (Radboud University Medical Center 2025). With these new numbers, the slopes of all three lines increased dramatically and exceeded those of their German counterparts for age groups 1 and 2 (Fig. 2). There was thus either a significant increase in relative trisomy costs C among Dutch women, or the decision-making process no longer followed the presumed decision theory model because age-specific prior risks were increasingly neglected.

The latter explanation seems rather likely because, whereas invasive testing was no longer offered to Dutch women ≥ 36 years from 2014 onwards, NIPT was introduced in the Netherlands in 2017 as a first-tier test that also covered age-independent structural chromosomal aberrations. This means that, while pregnant women also receive pre-test counselling in the Netherlands, maternal age has become a poorer predictor of a true-positive test result than in Germany and may hence no longer have played an important role in the counseling and decision-making of Dutch women about NIPT (van der Meij et al. 2019). In fact, in a 2018 survey of 600 Dutch women who underwent NIPT, only 1% of participants cited the perception of a high trisomy 21 risk as their main reason to do so (van der Meij et al. 2019; Meij et al. 2022).

Regardless of the plausibility of the above, an increase of the relative trisomy costs C could also apply, at least in part, to Dutch woman. In the quoted survey, 64% of participants thus expressed that they considered Down syndrome to be a serious condition, and 71% would have found it a great burden to raise a child with Down syndrome (van der Meij et al. 2022). Finally, NIPT is fully reimbursed in the Netherlands since April 2023 so that the test costs C_N_ decreased, and the relative costs C = C_T_/C_N_ increased, in all age groups.

In conclusion, the current NIPT uptake in Germany corresponds well to the age-specific prior risks of trisomies 21, 18 and 13, i.e. the three aneuploidies for which the test costs are reimbursed by the statutory health insurance. The validity of this claim is supported by its lack of transferability to the Netherlands, a country with a fairly similar cultural background to Germany, but with a clear violation of the underlying decision theory model due to the inherent independence of decision-making from the respective prior risks. Therefore, the political discussion on NIPT in Germany should refrain from implying that the test is recommended to pregnant women regardless of medical relevance which, in Germany, relates mainly to advanced maternal age. Replication of our analysis in other countries will reveal whether the consistency between age-specific prior risk and NIPT uptake observed in Germany is unique, or whether it also exists in other healthcare systems. Furthermore, we recommend continuous monitoring of the uptake in Germany itself to determine whether the current age-specific pattern is a temporary feature of the NIPT implementation phase or a more permanent feature of the national context.

## Data Availability

No datasets were generated or analysed during the current study.
